# Modelling of human Wiskott–Aldrich syndrome protein mutants in zebrafish larvae using in vivo live imaging 

**DOI:** 10.1242/jcs.128728

**Published:** 2013-09-15

**Authors:** Rebecca A. Jones, Yi Feng, Austen J. Worth, Adrian J. Thrasher, Siobhan O. Burns, Paul Martin

**Affiliations:** 1 School of Biochemistry, Faculty of Medical and Veterinary Sciences, University of Bristol, University Walk, Bristol, BS8 1TD, UK; 2 MRC Centre for Inflammation Research, Queen's Medical Research Institute, University of Edinburgh, 47 Little France Crescent, Edinburgh, EH16 4TJ, UK; 3 Molecular Immunology Unit, Institute of Child Health, University College London, 30 Guilford Street, London, WC1N 1EH, UK; 4 Great Ormond Street Hospital NHS Trust, Great Ormond Street, London, WC1N 3JH, UK; 5 Department of Immunology, Royal Free Hospital, University College London, Rowland Hill Street, Hampstead, London, NW3 2PF, UK

**Keywords:** Live imaging, Disease model, WASp, Zebrafish, Immune deficiency, Neutrophils, Macrophages

## Abstract

Wiskott–Aldrich syndrome (WAS) and X-linked neutropenia (XLN) are immunodeficiencies in which the function of several haematopoietic cell lineages is perturbed as a result of mutations in the actin regulator WASp. From *in vitro* cell biology experiments, and biochemical and structural approaches, we know much about the functional domains of WASp and how WASp might regulate the dynamic actin cytoskeleton downstream of activators such as Cdc42, but *in vivo* experiments are much more challenging. In patients, there is a correlation between clinical disease and genotype, with severe reductions in WASp expression or function associating with complex multilineage immunodeficiency, whereas specific mutations that cause constitutive activation of WASp result in congenital neutropenia. Here, we take advantage of the genetic tractability and translucency of zebrafish larvae to first characterise how a null mutant in zfWASp influences the behaviour of neutrophils and macrophages in response to tissue damage and to clearance of infections. We then use this mutant background to study how leukocyte lineage-specific transgenic replacement with human WASp variants (including normal wild type and point mutations that either fail to bind Cdc42 or cannot be phosphorylated, and a constitutively active mutant equivalent to that seen in XLN patients) alter the capacity for generation of neutrophils, their chemotactic response to wounds and the phagocytic clearance capacity of macrophages. This model provides a unique insight into WASp-related immunodeficiency at both a cellular and whole organism level.

## Introduction

Cells of the innate immune system depend on a precisely regulated actin cytoskeleton to drive a coordinated response to tissue damage and infection and to clear pathogens at sites of infection. From cell surface receptor through to actin nucleator, each component step in cytoskeletal regulation plays a pivotal role in enabling normal immune cell function.

Wiskott–Aldrich syndrome (WAS) protein WASp coordinates actin polymerisation via the Arp2/3 complex downstream of Cdc42 and kinase phosphorylation. Innate immune cells deficient in WASp, including neutrophils and macrophages, have been shown to display abnormal chemotaxis, motility and phagocytosis *in vitro* (Thrasher and Burns, 2010; Tsuboi and Meerloo, 2007). For example, tissue culture studies reveal that WASp-deficient macrophages fail to respond to chemotactic cues due to reduced persistence of directed protrusions (Ishihara et al., 2012; Zicha et al., 1998). They are also defective at phagocytosis of bacteria and apoptotic cells (Leverrier et al., 2001; Lorenzi et al., 2000).

Several studies indicate a correlation between the clinical phenotype of WAS and the nature of the inherited mutation (Jin et al., 2004), with truncated or abolished WASp expression coinciding with the most severe cases (Ochs and Thrasher, 2006). In contrast, X-linked Neutropenia (XLN) in patients, results from constitutively active mutations in WASp, and presents with congenital neutropenia (Ancliff et al., 2006; Devriendt et al., 2001).

In recent years zebrafish, *Danio rerio*, has come to the fore as a model for live imaging innate immune cell ontogeny, developmental homing and response to tissue damage, infection and cancer (Deng et al., 2012; Feng et al., 2010; Le Guyader et al., 2008; Levraud et al., 2009; Mathias et al., 2006; Niethammer et al., 2009; Prajsnar et al., 2008; Renshaw et al., 2006).

We have shown that the WASp protein is conserved in zebrafish and using a morpholino knockdown approach we previously reported that WASp deficient neutrophils and macrophages exhibit defects in their capacity to migrate towards a fin wound, suggesting that WASp function is conserved in fish (Cvejic et al., 2008). In this study we characterise a null mutant of zebrafish WASp, which shows defects in both the wound-induced inflammatory response and in immune-cell-mediated resistance to bacterial infection, thus mimicking the symptoms of human WAS patients. We then use transgenic replacement with a range of human WASp mutants using the Gal4:UAS system in order to observe how various human mutations affect innate immune cell behaviour *in vivo*.

## Results and Discussion

**Fig. 1.**
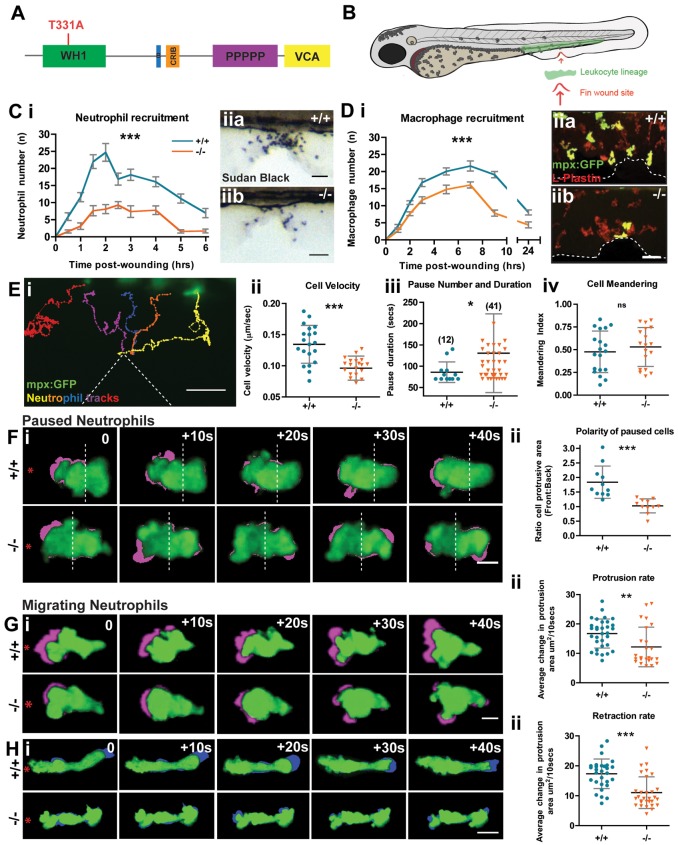
**The Zebrafish WASp mutant has a defect in leukocyte wound recruitment.** (A) Schematic of WASp protein domains with the site of the STOP codon in was^hu3280^ mutant indicated (red). (B) Schematic of 3 dpf zebrafish larva showing haematopoietic cell location (green) and wound (red arrow). (C) Time course of Sudan-Black-positive neutrophil recruitment in WT versus mutant larvae (i). Two-way ANOVA, Bonferroni post-test at each time-point 0.5 hour (NS), 1 hour (***), 1.5 hours (***), 2 hours (***), 2.5 hours (**), 3 hours (***), 4 hours (**), 5 hours (***), 6 hours (NS). Representative images of Sudan-Black-stained neutrophils recruited to the 90 minute wound site (ii) in WT (a) and mutant (b). (D) Time-course of macrophage recruitment in WT versus mutant mpx:GFP^+^ larvae (i). Two-way ANOVA, Bonferroni post-test at each time point 1 hour (NS), 2 hours (*), 3 hours (**), 5 hours (***), 7 hours (***), 9 hours (***), 24 hours (NS). Representative images of 3 hour wounds immunostained for L-Plastin to reveal all leukocytes (red) and mpx:GFP^+^ neutrophils (green) at the wound site (ii) in WT (a) and mutant (b). (E) Representative tracking analysis of mpx:GFP^+^ neutrophils during the first 90 minutes post wounding of a WT larva (i); each neutrophil track is in a different colour and the wound is indicated by dotted lines. Graphic representation of the velocity of migrating cells (µm/second) (ii), pause duration (seconds) with number of pauses >1 minute during wound migration (in brackets) (iii) and cell meandering index (iv), taken from tracks of WT (*n* = 20) and mutant (*n* = 20) mpx:GFP^+^ neutrophils. (F–H) Sample still images from confocal movies to illustrate protrusion/retraction analysis (i); neutrophils are green and new protrusive areas are indicated in magenta (F,G); and the retracting uropod in blue (H). WT cells exhibit greater persistence of polarity towards the wound (red asterisk). (F) (ii) Comparison of protrusive area (front versus back ratio analysis) in paused cells of WT vs mutant larvae. (G) (ii) As in F, but for migrating WT versus mutant cells, showing reduced protrusion in mutant cells. (H) (ii) Retraction analysis applied to migrating WT versus mutant cells, revealing mutant has defect in rate of uropod retraction. Error bars: s.e.m. (C,D); s.d. (E–H); *P<0.05; **P<0.01; ***P<0.001 by one-way ANOVA (C,D) and Student's *t*-test (E–H). NS, not significant. Scale bars: 10 µm (C,D); 20 µm (E); 5 µm (F–H).

Because zebrafish larvae are translucent we are able to live image and characterise the behaviour of WASp deficient innate immune cells in an *in vivo* context. We first characterise leukocyte behaviour in a zebrafish WASp null mutant (Fig. 1A) derived by TILLing (Cvejic et al., 2008), which we then use as a background line to investigate a series of human WASp alleles.

### The WASp mutant has normal numbers of leukocytes but they are defective in their ability to migrate to wounds

In humans, WASp is a haematopoietic cell restricted protein. We show that at the mRNA and protein levels, zebrafish WASp1 expression coincides with the leukocyte marker Lysozyme C (supplementary material Fig. S1A,B). We also confirm that WASp1 mutant larvae have normal numbers and distribution of neutrophils and macrophages (supplementary material Fig. S1C,D), just as observed in most human WAS patients. However, our wounding studies revealed reduced recruitment of both lineages at all time points from 30 minutes to 24 hours (Fig. 1C,D). Tracking analysis of mpx:GFP^+^ neutrophil migration in WASp1 mutants indicated increased frequency of ‘pausing’ and decreased cell velocity during migratory periods, but no significant increase in ‘meandering’ of tracks to the wound (Fig. 1E). We show that these alterations in migration are, at least in part, due to inefficiency in forming and maintaining new leading pseudopods in both paused and migrating cells, en route to wounds (Fig. 1F,G). WASp null cells also exhibited a reduced level of uropod retraction as they migrate towards the wound (Fig. 1H). We presume that all of these defects in cell migration to a wound are due to WASp-mediated influences on the cytoskeleton, but WASp proteins can also regulate endocytosis and there is precedent for endocytosis to dramatically influence the reading of chemotactic signals and so we cannot rule out this possibility (Jékely et al., 2005). There is a second WASp orthologue in zebrafish with less sequence homology to hWASp (Cvejic et al., 2008), but we saw no further migration defect after knockdown of this orthologue on a WASp mutant background (supplementary material Fig. S2).

### Dissecting the function of specific human WASp mutants in WASp null zebrafish

**Fig. 2.**
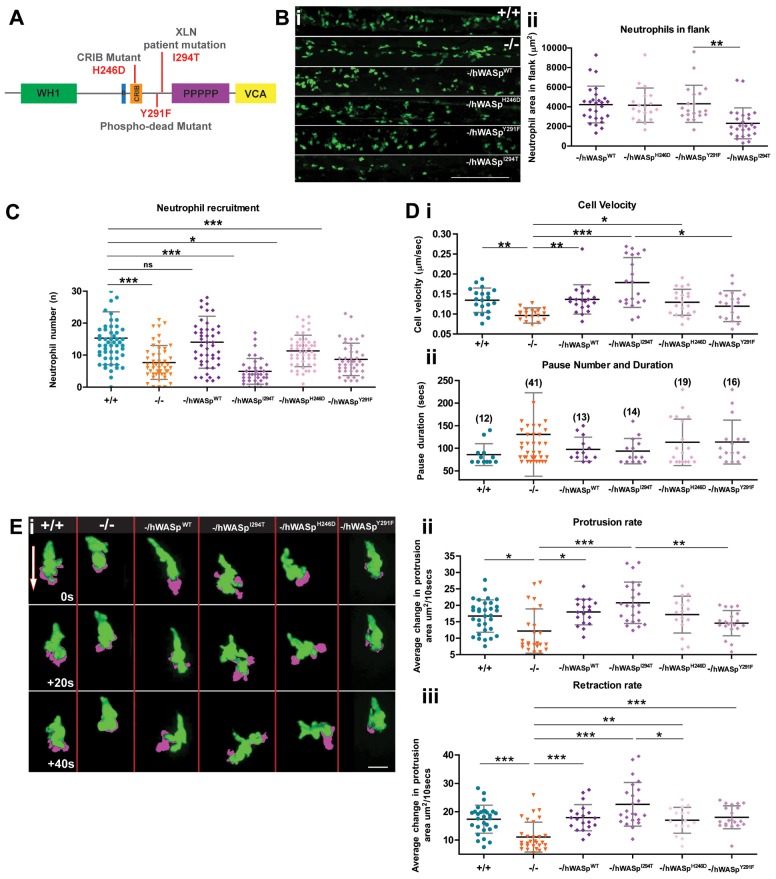
**The Zebrafish WASp mutant can be rescued to varying degrees by introduction of WT hWASp and clinical WASp mutants.** (A) Schematic of hWASp indicating the various mutant constructs for attempted rescue of the zebrafish mutant phenotype. (B) (i) lyz:Gal4-VP16 UAS:Kaede to reveal neutrophils (green), shows evidence of neutropenia only in the flanks of -/hWASp^I294T^ ‘rescued larvae’ with (ii) quantification of neutrophil deficiency by measurement of total neutrophil area in the hematopoietic region. (C) Degree of ‘rescue’ of neutrophil recruitment at 2 hours post wounding, after expression of hWASp constructs in the mutant background. (D) Tracking analysis of neutrophils following expression of each of the hWASp mutant constructs: (i) Quantification of the velocity of migrating cells (µm/sec), and (ii) pause duration (seconds), and pause number (in brackets). (E) Example still images from confocal time-lapse movies to illustrate protrusion analysis (magenta) applied to migrating hWASp mutant ‘rescues’ (see supplementary material Movie 1). Direction of the wound indicated by white arrow. (F) Quantification of rate of protrusion (i) and retraction (ii) in migrating hWASp mutant ‘rescues’. Error bars (s.d); asterisks denote significance values of *P<0.05, **P<0.01 and ***P<0.001 by one-way ANOVA. Scale bars: 100 µm (B); 10 µm (E).

Having confirmed that WASp1^−/−^ fish appear to model features of human WASp deficiency, we wondered whether hWASp might rescue these defects and whether we might use zebrafish larvae as a ‘window’ through which to observe cell behaviours in a range of hWASp lesions (Fig. 2A; supplementary material Movie 1). Immediately obvious, and mirroring the neutropenic phenotype of XLN patients, we found that expression of a constitutively active form of hWASp^I294T^ (Ancliff et al., 2006), using the neutrophil-specific Lysozyme C (lyz) promoter in otherwise WASp mutant fish, leads to a reduced overall number of neutrophils (Fig. 2B) in unwounded fish. We found that transgenic expression of full-length hWASp, again in otherwise WASp mutant fish, rescues both the numbers of neutrophils recruited to a wound (Fig. 2C) and their velocity and pause times en route to the wound (Fig. 2D). By contrast, transgenic expression of WASp^Y291F^, a phospho-dead mutant form of hWASp (Blundell et al., 2009), provided minimal improvement in neutrophil migration above the WASp mutant background (Fig. 2C), and this was mirrored in our movie analysis where we saw marginally slower migration of individual neutrophils (Fig. 2D) and concommitantly reduced protrusion dynamics (Fig. 2E). By contrast, when we expressed WASp^H246D^ in neutrophils, which cannot bind Cdc42 (Kato et al., 1999), we saw improvement in cell motility and recruitment to a level close to that in WT larvae (Fig. 2C) and normal protrusive outgrowth (Fig. 2E; supplementary material Movie 1) but with marginally reduced uropod retraction (Fig. 2E). Analysis of movies of the remaining neutrophils expressing the constitutively active form of hWASp^I294T^ (supplementary material Movie 1) showed these cells to be hyperprotrusive when responding to a wound, and with significantly increased velocity and increased protrusion and uropod retraction rates (Fig. 2D,E); we have seen a similar compensatory effect on migration in *Drosophila* macrophages mutant in Cdc42, which exhibited defects in cell polarisation, but with increased migratory velocity compared with WT cells (Stramer et al., 2005). We suggest that the more muted inflammatory response to wounds in these constitutively active WASp transgenic ‘rescues’ is largely due to the reduced number of responding neutrophils, rather than a retarded migratory capacity and this may reflect how the inflammatory process is also perturbed in XLN patients.

### The WASp mutant also models susceptibility to bacterial infection, just as in human patients

**Fig. 3.**
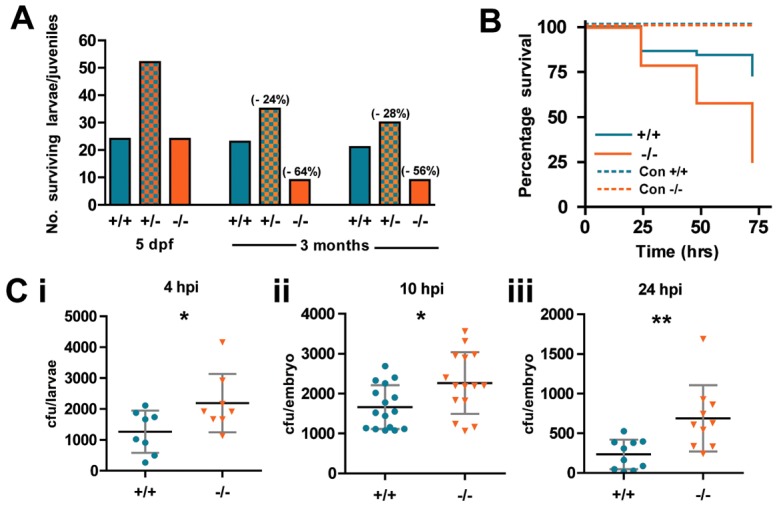
**WASp mutant larvae show increased susceptibility to death from bacterial infection.** (A) Ratio of genotypes of the offspring from WASp heterozygous in-crossed adults at 5 dpf and 3 months. Values in brackets indicate percentage drop in survival from predicted Mendelian ratio for WASp^+/−^ and WASp^−/−^ larvae. (B) Survival curves for WT (*n* = 44) versus mutant (*n* = 43) zebrafish larvae injected at 52 hpf with *S. aureus*; control injection is with PBS, WT (*n* = 15) mutant (*n* = 18). (C) Colony-forming unit (cfu) counts of individual larvae injected with 1200 cfu of *S. aureus* at (i) 4 hours post injection (hpi), WT (*n* = 8), mutant (*n* = 8); (ii) 10 hpi, WT (*n* = 16), mutant (*n* = 15); (iii) 24 hpi, WT (*n* = 10), mutant (*n* = 10). Error bars (s.d); asterisks denote significance values of *P<0.05, **P<0.01 using Student's *t*-test.

Patients with WAS and XLN are prone to bacterial infections. We observed that WASp^−/−^ juveniles are more prone to opportunistic bacterial infection and subsequent death (Fig. 3A). To investigate the degree of resistance of WASp mutants to bacterial infection, we challenged larvae with a systemic *S. aureus* infection, injected into the circulation at 52 hpf, at a dose previously shown to cause staggered larval death over 72 hours post injection (hpi) (Prajsnar et al., 2008). This revealed increased susceptibility to bacterial infection in WASp mutants (Fig. 3B) and greater bacterial burden (Fig. 3C).

**Fig. 4.**
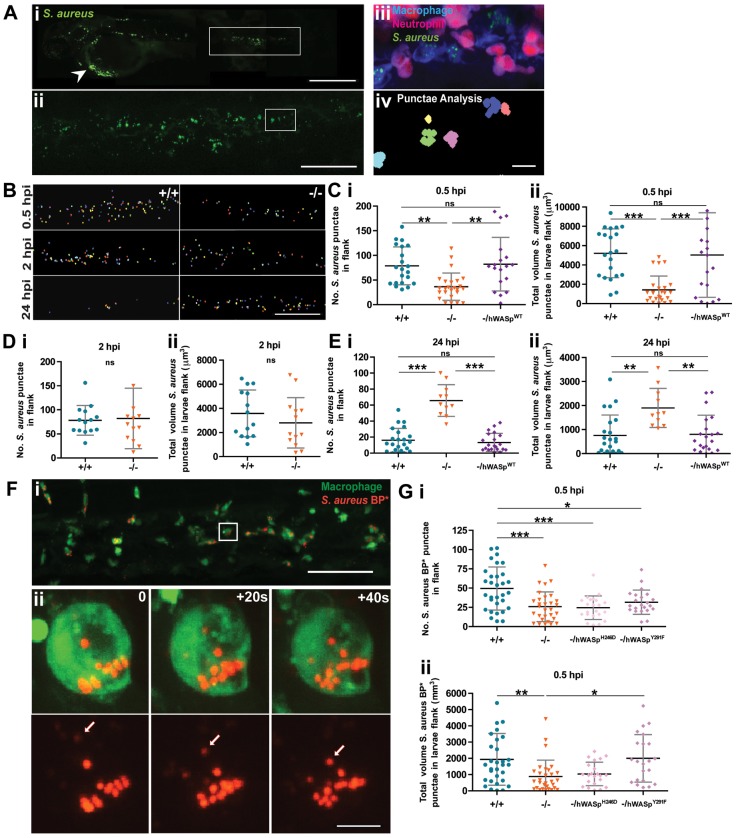
**WASp mutant larvae show a delay in phagocytosis and defective clearance of *S. aureus*.** (A) (i) Representative image of an infected larva. Arrowhead indicates injection site. (ii) GFP^+^ clumps of phagocytosed *S. aureus* visible in flank region (corresponding to white rectangle in i). (iii) Further magnification (corresponding to white square in ii) reveals a cluster of macrophages (blue) and neutrophils (magenta) with phagocytosed *S. aureus* (green) visible only within the macrophage population; (iv) application of punctae analysis using Volocity enables quantification of phagocytosed (as opposed to individual, not yet engulfed) *S. aureus*. (B) Representative images of bacterial punctae analysis in flanks of WT (+/+) versus mutant (−/−) larvae, revealing a delay in initial engulfment at 0.5 hpi, catch up by 2 hpi, but eventual failure to clear the phagocytosed bacteria at 24 hpi, in the mutant. (C–E) Scatter plots of (i) numbers of *S. aureus* punctae and (ii) total volume of *S. aureus* punctae (µm^3^) in flanks of WT versus mutant larvae at. Successful rescue of mutant defect at (C) 0.5 hpi and (E) 24 hpi following macrophage-specific re-expression of hWASp^WT^ under csf1a:Gal4. (F) (i) Confocal image of PU1:GFP-positive macrophages (green) in the haematopoietic region of 2 dpf larva 30 minutes post injection of pHrodo *S. aureus* Bioparticles (red). (ii) Stills from a time-lapse movie, illustrating increasing fluorescence of a single pHrodo bioparticle following acidification in the macrophage phagosome (see supplementary material Movie 3). Arrows in single channel series indicate phagosome as it becomes acidified. (G) Scatter plots of numbers of pHrodo *S. aureus* bioparticle punctae (i), and total volume of punctae (µm3) (ii), in flanks of WT, mutant and hWASp mutant ‘rescued’ larvae at 0.5 hpi. Error bars indicate s.d.; *P<0.05, **P<0.01 and ***P<0.001 via Student's *t*-test (D) and one-way ANOVA (C,E,G). Scale bars: 200 µm (Ai); 100 µm (Aii,B); 10 µm (Aiii); 50 µm (Fi); 5 µm (Fii).

In control WT fish, live imaging showed that most of the phagocytosis of circulating microbes, is accomplished by macrophages (Fig. 4A) (Colucci-Guyon et al., 2011) and commences almost immediately upon loading the circulation with *S. aureus* (supplementary material Movie 2). Using a ‘punctae analysis’ image quantification technique, we observed a clear delay in phagocytosis after 30 minutes in WASp mutant larvae, leading to a significant defect in clearance by 24 hpi (Fig. 4B–E) which explains their increased larval death rate. We saw no additional defect when we used morpholino knockdown WASp2 (data not shown). We found that transgenic expression of full-length hWASp in macrophages, using a csf1a promoter, in otherwise WASp mutant fish, rescues phagocytic uptake of microbes at both these timepoints to levels approaching WT (Fig. 4C,E). We next developed a more direct assay of phagocytosis without infection, utilising bioparticles that fluoresce only on acidification post phagocytic uptake (Fig. 4F; supplementary material Movie 3) (Brubaker et al., 2013). We used this assay to test the capacity of two of the hWASp mutant alleles investigated in our neutrophil motility ‘rescue’, but this time using the PU.1 promoter to drive them in macrophages. Our data here suggest a significantly increased ‘rescue’ of phagocytosis in cells expressing WASp^Y291F^, the phospho-dead mutant form of hWASp, above the WASp null background (Fig. 4G), whereas Cdc42 signalling seems absolutely essential because when we expressed WASp^H246D^, which cannot bind Cdc42, in otherwise WASp null macrophages, we saw no improvement in particle uptake (Fig. 4G).

This disassociation of Cdc42 activation and phosphorylation status of WASp has been reported previously and there is precedent from *in vitro* studies that WASp phosphorylation can occur independently of Cdc42 binding in cell migration and other WASp-dependent functions, whereas Cdc42 is essential for WASp-regulated phagocytosis (Badour et al., 2004; Cory et al., 2002; Park and Cox, 2009).

Overall, our data establish a model for investigating WASp function *in vivo* and reveal the potential for live imaging the effects of various human WAS disease-related mutations using zebrafish larvae as host ‘windows’ for observing immune cell behaviours. To our knowledge, this is the first occasion where a range of human disease alleles have been transgenically expressed and comparatively live imaged using zebrafish larvae in this way.

## Materials and Methods

### Zebrafish lines and maintenance

Adult zebrafish were maintained in standard conditions; breeding and genotyping were carried out according to standard protocols (Westerfield, 2000). Embryos were maintained at 28°C in 0.3× Danieu's solution. For strains generated and used in this study, see supplementary material Table S1.

### Leukocyte staining

Sudan Black staining was used to reveal neutrophils (Cvejic et al., 2008), and a pan-leukocytic antibody, L-Plastin was used to stain for all leukocytes as previously described (Redd et al., 2006).

### Tail-fin wounding

Mechanical wounding was performed as previously described (Cvejic et al., 2008); in brief, 3 dpf larvae were anaesthetised and their ventral tail fin wounded using a tungsten needle (Fig. 1B).

### Infection protocol

1 nl of 1.2×10^9^ cfu ml^−1^ GFP-expressing *S. aureus* or 2 mg/ml pHrodo-*S. aureus* BioPaticles (Invitrogen), resuspended in PBS, were microinjected into the Duct of Cuvier of anaesthetised 52 dpf larvae. For live *S. aureus* injections, WT and mutant larvae were monitored over the course of 3 days until 5 dpf. Dead larvae were counted and removed at 24 hour post injection (hpi), 48 hpi and 72 hpi. To measure bacterial burden, individual 4 hpi, 10 hpi and 24 hpi larvae were processed for cfu analysis using an adaption of a previous protocol (Prajsnar et al., 2008), whereby each larva was first lysed in 400 ul 0.5% Triton X-100 and plated on a 90 mm LB agar plate.

### *In vivo* live imaging

Live imaging was performed according to our previously published protocol. (Cvejic et al., 2008). A widefield Leica DMIRB inverted microscope was used to take movies for tracking studies and an Ultraview Spinning Disk Confocal imaging system with a 63× glycerol lens was used for protrusion/retraction analysis.

### Image acquisition and quantification

Quantification of phagocytosed bacterial/BioParticle burden was performed on images of fixed, infected larval flanks, captured on a confocal microscope using 20× lens. Images were processed using Volocity (Improvision) software with GFP^+^/RFP^+^ bacterial clumps identified using s.d. intensity (12<s.d.<170). Parameters were refined to isolate large phagosomes over individual bacterium/BioParticles by setting a size threshold >5 µm. The manual tracking function of Volocity 5.0 (Improvision) was used to track neutrophil migration. Quantification of new protrusive areas and uropod retraction was performed using NIH ImageJ 1.42q software. On conversion of the movie to 8-bit greyscale, the movie was duplicated. In the duplicate copy the first frame was removed and a blank frame added at the end. Using the ‘Image calculator’ function the duplicate movie was subtracted from the original to reveal the new protrusive area; the reverse process was applied for retraction analysis. Following inversion of the image, the protrusive/retracted areas (µm^2^) could be calculated using the function ‘Analyse particles’. For paused cell movie analysis, the cell was divided in half, front and back, using the location of wound as a reference point, before calculation of areas were performed.

### Construction of vectors for generating transgenic fish for each hWASp mutant

cDNAs for each eGFP-WASp mutant were cloned in frame between (NheI, ClaI) sites of the transgenic parental vector pBH-UASmsc, which contains the Tol2 sequence and a *mlc* promoter driven mCherry as transgene screen marker (kindly provided by Michael Nonet, Washington University). All the constructs were sequenced to verify their coding sequences.

### Establishing zebafish transgenic strains containing UAS-WASp mutants

A Tol2 transposase mediated transgenic strategy (Kawakami et al., 2000) was used to generate UAS-WASp mutant strains following the established protocol (Balciunas et al., 2006). F0 embryos were grown to adulthood and then either in crossed or crossed to WASp^−/−^; their offspring were screened using fluorescent red heart as transgenic marker. F1 embryos with red hearts were grown up to establish Tg(BH-UAS-mcs-hWASp-eGFP); Tg(BH-UAS-mcs-hWASp^A134T^-eGFP); Tg(BH-UAS-mcs-hWASp^H246D^-eGFP); Tg(BH-UAS-mcs-hWASp^Y291F^-eGFP); Tg(BH-UAS-mcs-hWASp^I294T^-eGFP), that were used in this study and crossed as appropriate.

For each of our rescue experiments we have tested, as far as is possible, whether physiologically relevant levels of transgenic hWASp mRNAs/proteins are being expressed, by semi-quantitive RT-PCR (bands of similar intensity in WT and rescued larvae, supplementary material Fig. S1E), and immunostaining (data not shown), although absolute comparisons are not possible because we use different antibodies to image the zebrafish and human WASp proteins.

### Production of WASp1 antibody

Zebrafish WASp1 cDNA fragment (1–699) was cloned into pGEX in frame. pGEX-zfWASp (1–699) was used to express a zfWASp–GST fusion protein in *E. coli* BL21 following the standard manufacturer's protocol. zfWASp was affinity purified using a Gluthathione Sepharose 4B column, and the final protein was sent to David's Biotechnologie (Germany) for production of rabbit polyclonal antibody. The specificity of the received zfWASp1 anti-serum was confirmed by ELISA.

### WASp2 morpholino

Splice blocking (CAGAGACTGCAGACAAAAACACAAA) and translation blocking (CTTTCCCCTTCCGGCTCGCCTCAT) morpholinos were designed against WASp2 (Cvejic et al., 2008) and injected at concentration of 2 µg/µl and 2.5 µg/µl, respectively.

### *In situ* probes

zfWASp1 cDNA was cloned into pTOPOCRII (Invitrogen). *Not*I enzyme was used to linearise the vector and SP6 was used to transcribe DIG labeled RNA probe for zfWASp1 using a Clontech DIG RNA labelling mix and following the manufacturer's guidelines.

### RT-PCR and qPCR

Ten larvae from each genotype were lysed in 1 ml TRIzol (Invitrogen) and RNA were purified following the manufacturer's user guide. First strand cDNA were synthesised using SuperScript III Reverse Transcriptase system (Invitrogen), following the user manual and resulting cDNAs were used for subsequent PCR assays. qPCR was performed using an Opticon2 machine and SYBR green reagents, according to the manufacturer's instructions. Primer sequences used were as follows (5′–3′): zfWASp1 forward (CAGTTATACATGGCCCTTCCTC), reverse (CACTTTCGCTTCCATAATGTCA); L-Plastin forward (CCTGACGGATGAAAAGAAGC), reverse (ATCACCATCTTGGGCTTCAC); hWASp forward (TACTTCATCCGCCTTTACGG), reverse (GTCATCTCCAGCGAAGGTGT); Efα (CTGGTTCAAGGGATGGAAGA), reverse (GAGACTCGTGGTGCATCTCA). For all the reactions, annealing temperature was 60°C, with elongation for 30 seconds, for 40 cycles.

## References

[b1] Ancliff P. J. Blundell M. P. Cory G. O. Calle Y. Worth A. Kempski H. Burns S. Jones G. E. Sinclair J. Kinnon C. (2006). Two novel activating mutations in the Wiskott-Aldrich syndrome protein result in congenital neutropenia. Blood 108, 2182–2189. 10.1182/blood-2006-01-010249 16804117

[b2] Badour K. Zhang J. Shi F. Leng Y. Collins M. Siminovitch K. A. (2004). Fyn and PTP-PEST-mediated regulation of Wiskott-Aldrich syndrome protein (WASp) tyrosine phosphorylation is required for coupling T cell antigen receptor engagement to WASp effector function and T cell activation. J. Exp. Med. 199, 99–112. 10.1084/jem.20030976 14707117 PMC1887720

[b3] Balciunas D. Wangensteen K. J. Wilber A. Bell J. Geurts A. Sivasubbu S. Wang X. Hackett P. B. Largaespada D. A. McIvor R. S. (2006). Harnessing a high cargo-capacity transposon for genetic applications in vertebrates. PLoS Genet. 2, e169 10.1371/journal.pgen.0020169 17096595 PMC1635535

[b4] Blundell M. P. Bouma G. Metelo J. Worth A. Calle Y. Cowell L. A. Westerberg L. S. Moulding D. A. Mirando S. Kinnon C. (2009). Phosphorylation of WASp is a key regulator of activity and stability in vivo. Proc. Natl. Acad. Sci. USA 106, 15738–15743. 10.1073/pnas.0904346106 19805221 PMC2736139

[b5] Brubaker A. L. Rendon J. L. Ramirez L. Choudhry M. A. Kovacs E. J. (2013). Reduced neutrophil chemotaxis and infiltration contributes to delayed resolution of cutaneous wound infection with advanced age. J. Immunol. 190, 1746–1757. 10.4049/jimmunol.1201213 23319733 PMC3563860

[b6] Colucci-Guyon E. Tinevez J. Y. Renshaw S. A. Herbomel P. (2011). Strategies of professional phagocytes in vivo: unlike macrophages, neutrophils engulf only surface-associated microbes. J. Cell Sci. 124, 3053–3059. 10.1242/jcs.082792 21868367

[b7] Cory G. O. Garg R. Cramer R. Ridley A. J. (2002). Phosphorylation of tyrosine 291 enhances the ability of WASp to stimulate actin polymerization and filopodium formation. Wiskott-Aldrich Syndrome protein. J. Biol. Chem. 277, 45115–45121. 10.1074/jbc.M203346200 12235133

[b8] Cvejic A. Hall C. Bak-Maier M. Flores M. V. Crosier P. Redd M. J. Martin P. (2008). Analysis of WASp function during the wound inflammatory response—live-imaging studies in zebrafish larvae. J. Cell Sci. 121, 3196–3206. 10.1242/jcs.032235 18782862

[b9] Deng Q. Harvie E. A. Huttenlocher A. (2012). Distinct signalling mechanisms mediate neutrophil attraction to bacterial infection and tissue injury. Cell. Microbiol. 14, 517–528. 10.1111/j.1462-5822.2011.01738.x 22188170 PMC3302966

[b10] Devriendt K. Kim A. S. Mathijs G. Frints S. G. M. Schwartz M. Van Den Oord J. J. Verhoef G. E. G. Boogaerts M. A. Fryns J. P. You D. Q. (2001). Constitutively activating mutation in WASP causes X-linked severe congenital neutropenia. Nat. Genet. 27, 313–317. 10.1038/85886 11242115

[b11] Elks P. M. van Eeden F. J. Dixon G. Wang X. Reyes-Aldasoro C. C. Ingham P. W. Whyte M. K. Walmsley S. R. Renshaw S. A. (2012). Activation of hypoxia-inducible factor-1α (Hif-1α) delays inflammation resolution by reducing neutrophil apoptosis and reverse migration in a zebrafish inflammation model. Blood 118, 712–722. 10.1182/blood-2010-12-324186 21555741

[b12] Feng Y. Santoriello C. Mione M. Hurlstone A. Martin P. (2010). Live imaging of innate immune cell sensing of transformed cells in zebrafish larvae: parallels between tumor initiation and wound inflammation. PLoS Biol. 8, e1000562 10.1371/journal.pbio.1000562 21179501 PMC3001901

[b13] Gray C. Loynes C. A. Whyte M. K. Crossman D. C. Renshaw S. A. Chico T. J. (2011). Simultaneous intravital imaging of macrophage and neutrophil behaviour during inflammation using a novel transgenic zebrafish. Thromb. Haemost. 105, 811–819. 10.1160/TH10-08-0525 21225092

[b14] Hall C. Flores M. V. Storm T. Crosier K. Crosier P. (2007). The zebrafish lysozyme C promoter drives myeloid-specific expression in transgenic fish. BMC Dev. Biol. 7, 42 10.1186/1471-213X-7-42 17477879 PMC1877083

[b15] Hatta K. Tsujii H. Omura T. (2006). Cell tracking using a photoconvertible fluorescent protein. Nat. Protoc. 1, 960–967. 10.1038/nprot.2006.96 17406330

[b16] Hsu K. Traver D. Kutok J. L. Hagen A. Liu T. X. Paw B. H. Rhodes J. Berman J. N. Zon L. I. Kanki J. P. (2004). The pu.1 promoter drives myeloid gene expression in zebrafish. Blood 104, 1291–1297. 10.1182/blood-2003-09-3105 14996705

[b17] Ishihara D. Dovas A. Park H. Isaac B. M. Cox D. (2012). The chemotactic defect in wiskott-Aldrich syndrome macrophages is due to the reduced persistence of directional protrusions. PLoS ONE 7, e30033 10.1371/journal.pone.0030033 22279563 PMC3261183

[b18] Jékely G. Sung H. H. Luque C. M. Rørth P. (2005). Regulators of endocytosis maintain localized receptor tyrosine kinase signaling in guided migration. Dev. Cell 9, 197–207. 10.1016/j.devcel.2005.06.004 16054027

[b19] Jin Y. Z. Mazza C. Christie J. R. Giliani S. Fiorini M. Mella P. Gandellini F. Stewart D. M. Zhu Q. L. Nelson D. L. (2004). Mutations of the Wiskott-Aldrich Syndrome Protein (WASP): hotspots, effect on transcription, and translation and phenotype/genotype correlation. Blood 104, 4010–4019. 10.1182/blood-2003-05-1592 15284122

[b20] Kato M. Miki H. Imai K. Nonoyama S. Suzuki T. Sasakawa C. Takenawa T. (1999). Wiskott-Aldrich syndrome protein induces actin clustering without direct binding to Cdc42. J. Biol. Chem. 274, 27225–27230. 10.1074/jbc.274.38.27225 10480940

[b21] Kawakami K. Shima A. Kawakami N. (2000). Identification of a functional transposase of the Tol2 element, an Ac-like element from the Japanese medaka fish, and its transposition in the zebrafish germ lineage. Proc. Natl. Acad. Sci. USA 97, 11403–11408. 10.1073/pnas.97.21.11403 11027340 PMC17212

[b22] Le Guyader D. Redd M. J. Colucci-Guyon E. Murayama E. Kissa K. Briolat V. Mordelet E. Zapata A. Shinomiya H. Herbomel P. (2008). Origins and unconventional behavior of neutrophils in developing zebrafish. Blood 111, 132–141. 10.1182/blood-2007-06-095398 17875807

[b23] Leverrier Y. Lorenzi R. Blundell M. P. Brickell P. Kinnon C. Ridley A. J. Thrasher A. J. (2001). Cutting edge: the Wiskott-Aldrich syndrome protein is required for efficient phagocytosis of apoptotic cells. J. Immunol. 166, 4831–4834. 11290758 10.4049/jimmunol.166.8.4831

[b24] Levraud J. P. Disson O. Kissa K. Bonne I. Cossart P. Herbomel P. Lecuit M. (2009). Real-time observation of listeria monocytogenes-phagocyte interactions in living zebrafish larvae. Infect. Immun. 77, 3651–3660. 10.1128/IAI.00408-09 19546195 PMC2738018

[b25] Lorenzi R. Brickell P. M. Katz D. R. Kinnon C. Thrasher A. J. (2000). Wiskott-Aldrich syndrome protein is necessary for efficient IgG-mediated phagocytosis. Blood 95, 2943–2946. 10779443

[b26] Mathias J. R. Perrin B. J. Liu T. X. Kanki J. Look A. T. Huttenlocher A. (2006). Resolution of inflammation by retrograde chemotaxis of neutrophils in transgenic zebrafish. J. Leukoc. Biol. 80, 1281–1288. 10.1189/jlb.0506346 16963624

[b27] Niethammer P. Grabher C. Look A. T. Mitchison T. J. (2009). A tissue-scale gradient of hydrogen peroxide mediates rapid wound detection in zebrafish. Nature 459, 996–999. 10.1038/nature08119 19494811 PMC2803098

[b28] Ochs H. D. Thrasher A. J. (2006). The Wiskott-Aldrich syndrome. J. Allergy Clin. Immunol. 117, 725–738.quiz 739 10.1016/j.jaci.2006.02.005 16630926

[b29] Park H. Cox D. (2009). Cdc42 regulates Fc gamma receptor-mediated phagocytosis through the activation and phosphorylation of Wiskott-Aldrich syndrome protein (WASP) and neural-WASP. Mol. Biol. Cell 20, 4500–4508. 10.1091/mbc.E09-03-0230 19741094 PMC2770938

[b30] Prajsnar T. K. Cunliffe V. T. Foster S. J. Renshaw S. A. (2008). A novel vertebrate model of Staphylococcus aureus infection reveals phagocyte-dependent resistance of zebrafish to non-host specialized pathogens. Cell. Microbiol. 10, 2312–2325. 10.1111/j.1462-5822.2008.01213.x 18715285

[b31] Redd M. J. Kelly G. Dunn G. Way M. Martin P. (2006). Imaging macrophage chemotaxis in vivo: studies of microtubule function in zebrafish wound inflammation. Cell Motil. Cytoskeleton 63, 415–422. 10.1002/cm.20133 16671106

[b32] Renshaw S. A. Loynes C. A. Trushell D. M. Elworthy S. Ingham P. W. Whyte M. K. (2006). A transgenic zebrafish model of neutrophilic inflammation. Blood 108, 3976–3978. 10.1182/blood-2006-05-024075 16926288

[b33] Stramer B. Wood W. Galko M. J. Redd M. J. Jacinto A. Parkhurst S. M. Martin P. (2005). Live imaging of wound inflammation in Drosophila embryos reveals key roles for small GTPases during in vivo cell migration. J. Cell Biol. 168, 567–573. 10.1083/jcb.200405120 15699212 PMC2171743

[b34] Thrasher A. J. Burns S. O. (2010). WASP: a key immunological multitasker. Nat. Rev. Immunol. 10, 182–192. 10.1038/nri2724 20182458

[b35] Tsuboi S. Meerloo J. (2007). Wiskott-Aldrich syndrome protein is a key regulator of the phagocytic cup formation in macrophages. J. Biol. Chem. 282, 34194–34203. 10.1074/jbc.M705999200 17890224

[b36] Westerfield M. (2000). The Zebrafish Book: A Guide to the Laboratory Use of Zebrafish (Danio rerio) 4th edn Eugene, OR: University of Oregon Press.

[b37] Zicha D. Allen W. E. Brickell P. M. Kinnon C. Dunn G. A. Jones G. E. Thrasher A. J. (1998). Chemotaxis of macrophages is abolished in the Wiskott-Aldrich syndrome. Br. J. Haematol. 101, 659–665. 10.1046/j.1365-2141.1998.00767.x 9674738

